# A Systematic Review and Lived Experience Synthesis of Self-disclosure as an Active Ingredient in Interventions for Adolescents and Young Adults with Anxiety and Depression

**DOI:** 10.1007/s10488-023-01253-2

**Published:** 2023-02-04

**Authors:** Pattie P. Gonsalves, Rithika Nair, Madhavi Roy, Sweta Pal, Daniel Michelson

**Affiliations:** 1 School of Psychology, University of Sussex, Brighton, UK; 2 Sangath, E-5, Lane 1, Westend Marg, Saiyad ul ajaib, Saket, New Delhi, 110030 India; 3 Department of Child and Adolescent Psychiatry, Institute of Psychiatry, Psychology and Neuroscience, King’s College London, London, UK

**Keywords:** Self-disclosure, Adolescents, Young adults, Depression, Anxiety, Systematic review

## Abstract

Self-disclosure, referring to the ability to communicate and share intimate personal feelings, has strong face validity for many young people as a way of improving anxiety and depression outcomes. The current review aimed to generate the first comprehensive evidence synthesis of self-disclosure interventions involving young people aged 14–24 years who are either disclosers or recipients of personal information about living with anxiety and/or depression. A systematic review of quantitative and qualitative data was combined with new insights from an adolescents and young adults lived-experience panel (n = 7) with the intention to combine rigorous systematic review methods and experiential knowledge. Six studies of variable quality were included in this review, five were quantitative and one was qualitative. Findings suggest that self-disclosure may be effective at reducing symptoms for adolescents and young adults with established depression; effects were not apparent when delivered as early prevention. No evidence for impacts on anxiety was found. The potential for negative effects like bullying or harassment was identified. Findings were limited by a small number of studies; low representation of peer-reviewed studies from low-or middle-income countries; and varied interventions in terms of format, participants' context, and nature of delivery. Self-disclosure may be of value in the context of interventions intended explicitly to reduce depression for those already showing symptoms. Delivery by non-specialists (such as peers and teachers) in addition to mental health professionals can help build capacity in community health systems. Self-disclosure may also be helpful at reducing stigma and stimulating help-seeking at earlier stages of mental health problems.

## Introduction

Anxiety and depression are the most prevalent mental health problems globally and affect one in five people, with peak onset occurring during adolescence and early adulthood (WHO), W.H.O. 2021; Solmi, 2021). Symptoms frequently co-occur, including mixed subsyndromal presentations that are common in the early stages of illness. Early interventions are key to improving long-term prognosis and mitigating potential negative impacts on interpersonal relationships, academic achievement, and future employment (Davey & McGorry, 2019; Salazar de Pablo, et al., 2021). Yet despite evidence for effective psychosocial interventions, most young people with anxiety or depression do not receive appropriate help, particularly in low-and middle-income countries which contain 90% of the world’s population aged under 25 years (Li et al., 2018; Patel et al., 2013; Yatham et al., 2018). Aside from well documented supply side barriers (i.e., due to human resource shortages), stigma continues to be a powerful demand-side barrier to mental health care (Radez et al., 2021; Scior et al., 2020). Even in high-income settings, mental health services are often over stretched or fragmented, meaning young people cannot access formal support and a growing number ‘fall between the gaps’ (Fusar-Poli et al., 2021).

Fundamental changes are required in service organisation and delivery to enhance capacity and align interventions with young people’s key concerns (Fusar-Poli et al., 2021; Gulliver et al., 2010; Persson et al., 2017). Looking beyond conventional therapist-led practice elements (i.e., discrete components of active preventive or therapeutic interventions), self-disclosure has strong face validity for many young people as a way of improving anxiety and depression outcomes (Kahn & Garrison, 2009; Kahn & Hessling, 2001). Referring to the ability to communicate and share intimate personal feelings (Cozby, 1973; Jourard & Lasakow, 1958), self-disclosure is conducted increasingly through online social networking in addition to ‘offline’ behaviours (Luo & Hancock, 2020; Vijayakumar & Pfeifer, 2020). It can take the form of verbal or written emotional expression whereby emotional experiences are articulated into words and communicated to others via written or spoken channels (Berry et al., 1998).

Ng et al., 2016 found that talking with supportive peers and family members was ranked second out of 20 evidence-based practice elements by depressed adolescents in terms of its perceived effectiveness and congruence with habitual coping strategies (i.e., fit with coping strategies that young people enact spontaneously). Other research shows that young people seek help through talking to their family and friends and may be more inclined to seek professional help if they feel able to express their feelings (Rickwood et al., 2007).

Several studies have documented the proximal impacts, including both benefits and potential harms, of young people ‘coming out’ or disclosing personal experiences of mental health problems. Positive impacts for the ‘disclosers’ include reduced self-stigma (Corrigan, 2012; Corrigan et al., 2016; Goodwin et al., 2021), improved quality of life and personal empowerment (Corrigan & Shapiro, 2010), and enhanced social support (Bos et al., 2009).

Potential harms of disclosure such as labelling and discrimination have additionally been documented (Greene et al., 2006; Pachankis, 2007), as well as individual variation in the extent to which some young people use self-disclosure habitually to manage their symptoms (Ng et al., 2016).

The effects of self-disclosure can also be understood from the perspective of young people who receive personally disclosed information from others, as occurs in social contact-based interventions (i.e., those involving interpersonal contact with individuals from stigmatised groups). Such interventions may be effective at reducing stigma and promoting help-seeking among ‘recipients’ (Corrigan, 2012; Thornicroft et al., 2016; Yanos et al., 2015). A common feature described in these interventions is a discloser with lived experience who describes pathways to attaining life goals while coping with mental health-related challenges (Corrigan et al., 2014, 2016). This may be accompanied by autobiographical information intended to disconfirm stereotypes, highlight adaptive coping strategies, and convey messages of recovery and hope for the recipients (Reinke, et al., 2004).

Distal impacts of self-disclosure on common mental health problems for adolescents and young adults have been less commonly studied and there is no published systematic review of relevant interventions. Thus, the current review aimed to generate the first comprehensive evidence synthesis of self-disclosure interventions involving young people aged 14–24 years who are either disclosers or recipients of personal information about living with anxiety and/or depression. We intended to create an inclusive synthesis involving published peer-reviewed research using any quantitative, qualitative, or mixed methodology. We also involved an adolescents and young adults lived experience advisory group in the process of evidence synthesis, with the intention to combine rigorous systematic review methods and experiential knowledge (Oliver et al., 2015; Sellars et al., 2021) in answering the following research questions:

1. What is the evidence for the benefits and potential harms of self-disclosure in interventions aimed at preventing, treating, or managing anxiety and depression among 14–24 years-olds?
2. For which clinical and demographic subpopulations does self-disclosure appear to be more/less effective?
3. In which contexts (including wider settings, frequency, and modes of delivery) do self-disclosure interventions appear to be more/less effective?
4. What are the putative mechanisms by which self-disclosure interventions influence outcomes in the target population?

## Methods

### Study Design and Research Questions

Given the focus of this review on impacts, experience, and mechanisms of self-disclosure interventions, we undertook a mixed method review of quantitative and qualitative data. The purpose was to combine evidence of ‘what works’ and ‘how and why it works’, thus offering a more comprehensive understanding (Grant & Booth, 2009).

### Protocol and Registration

This review was registered on PROSPERO on 6th September 2021 (CRD42021272033).

### Study Search

A systematic search was conducted in PubMed/Medline, PsycARTICLES, PsycINFO, Web of Science, CINAHL Plus and Cochrane library between the 9th and 11th August 2021 using terms reflecting the age range (14–24 years), self-disclosure, depression, anxiety, and research design. Full search terms are provided in Appendix A. The search was limited to peer-reviewed published studies, presenting primary data, and written in the English language. The date of publication was limited to studies from the year 2000 onwards. Screening and selection were managed using Covidence software (Innovation, 2021).

### Study Selection

Study inclusion criteria were informed by the SPIDER tool for qualitative/mixed methods research (Cooke et al., 2012). Identified references were screened according to the following criteria. First, interventions were focused on young people aged 14–24 years either as disclosers or as recipients of self-disclosure. Disclosers were required to have a current or past experiences of depression and/or anxiety based on (a) a clinical diagnosis made by a mental health professional, (b) elevated symptoms confirmed by a standardized assessment tool, or (c) subjective self-report. In the case of preventive interventions, recipients could be young people who have not (yet) experienced either condition. Second, interventions employed intentional self-disclosure, defined as revealing personal information about lived experiences of anxiety or depression, with the goal to prevent onset of, treat, manage, or prevent relapse of anxiety and/or depression for the disclosers and/or recipients of such information. Third, studies used qualitative, quantitative, or mixed method. Fourth, studies were conducted in any geographical location, and with no restrictions on health, community, educational or online setting. Fifth, primary quantitative outcomes of interest were improvements in depression and anxiety, measured by validated symptom-based or diagnostic instruments or qualitative reports of symptomatic/diagnostic and functional improvements. Full criteria are provided in Appendix B.

Study screening was conducted by four reviewers. Disagreements between reviewers were managed through consensus methods, or else taking the majority decision in the absence of full consensus.

### Data Extraction

Two researchers independently conducted data extraction using Excel. Extracted information on study characteristics included study design and methodology, the geographical location and setting, participant demographics and baseline characteristics, description of how self-disclosure was conducted, and data collection methods. Outcomes of interest were depression and anxiety, measured by validated symptom-based or diagnostic instruments and qualitative reports of symptomatic/diagnostic and functional improvements (e.g., in interpersonal, occupational, and educational domains). Additional outcomes were related to potential harms (deteriorations in depression and anxiety or qualitative reports of negative effects). Evidence on mechanisms was obtained from qualitative reports of how intervention content or materials were used by recipients/disclosers in interventions or descriptions or pathways to beneficial outcomes or potential harms.

### Lived Experience Panel

Seven Indian young adults aged 19–26 years of different genders (71% female) formed a lived experience panel referred to as the ‘Young People’s Advisory Group’ (YPAG). All members had lived experiences of anxiety and/or depression and most had accessed formal mental health services. The YPAG was recruited by invitation from existing networks of young people who had participated in mental health awareness and research activities through the ‘It’s Ok to Talk’ (Sangath, 2021) public engagement programme implemented by Sangath NGO. The YPAG participated in six separate two-hour long virtual meetings using Zoom video-conferencing software (Inc., Z.V.C. & Zoom., 2021) and with additional contributions elicited by email and WhatsApp (Meta, 2021). Participation was reimbursed through an honorarium provided for attendance at sessions and completion of self-work.

The YPAG was involved in three main activities: (i) individually commenting on the search terms for the review; (ii) contributing through group discussion to the interpretation of findings including identifying helpful and problematic aspects of candidate self-disclosure interventions and priorities for future research, and (iii) working in smaller groups to conduct an online search of publicly accessible self-disclosure projects focused on or led by young people via online channels like websites, blogs, or social media and through multi-media or arts projects not covered in peer-reviewed literature. This activity was aimed at encouraging the YPAG to engage with concepts, processes and potential impacts related to self-disclosure interventions (a summary of their findings is provided in Appendix C).

Thematic summaries of YPAG sessions and individual feedback were prepared by the research team and incorporated into the results.

### Risk of Bias

Risk of bias within each study was rated with the Mixed Methods Appraisal Tool (MMAT) (Hong et al., 2018), using the two filter questions and then five quantitative and/or qualitative criteria as appropriate.

### Synthesis of Results

A narrative evidence synthesis (Popay et al., 2006) was supplemented by insights generated from the YPAG activities. The PRISMA statement (Moher et al., 2010) was used to prepare this report. Within and between-group effect sizes/p-values and corresponding qualitative data were summarised and organised thematically around the core review questions. Verbatim quotes from the YPAG have been presented in italics.

## Results

### Study Selection

**Fig. 1:**
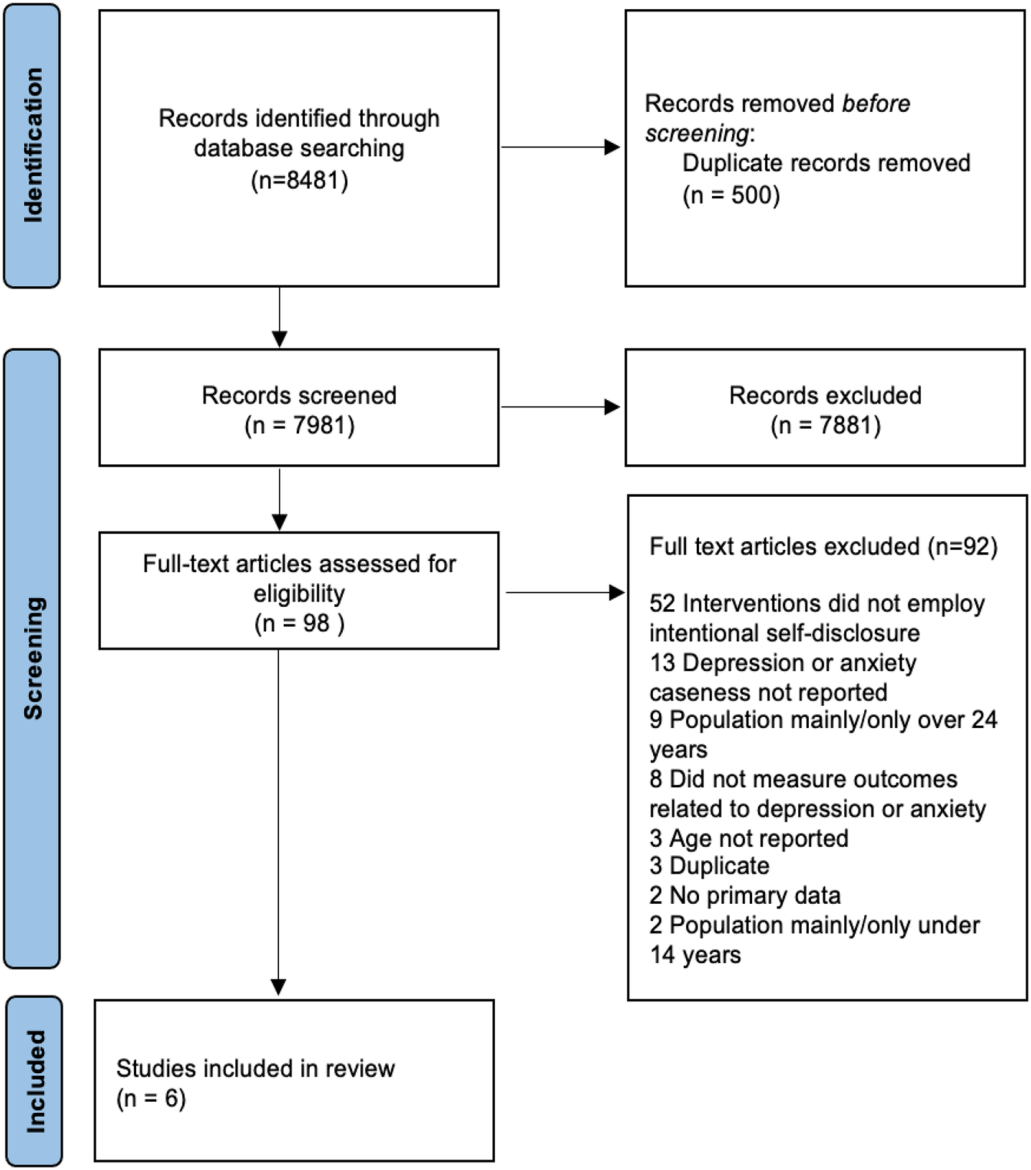
PRISMA diagram of study selection (Moher et al., 2010)

Four reviewers screened 7981 records (Fig. 1) using the titles and abstracts. Two authors screened all studies and two other reviewers independently screened 2049 records (25.6%). Reviewer agreement about eligibility was 93.5%. At the full text stage, all records were screened by two authors independently, with reviewer agreement at 68.4%.

### Study and Participant Characteristics

Six studies were included in this review (Table 1). Four studies were randomized controlled trials (RCTs), one was a follow-up study of one of the included RCTs, and one used a phenomenological qualitative design using interviews and focus-group discussions. Studies were conducted in Germany (n = 2), USA (n = 2), Nigeria (n = 1) and Sweden (n = 1). Most studies recruited participants from colleges (n = 2) or schools (n = 2), online crowdsourcing (n = 1) or clinics (n = 1). Study settings included schools (n = 2), universities (n = 2), web-based (n = 1) and clinics (n = 1). Of the school-based studies, one was conducted the study with student athletes with special education needs.

**Table 1:** Study and participant characteristics

Paper	Design	Total sample	Aim	Recruitment setting	Participant eligibility		Mean age (SD), Range	Gender %
					Population	Severity at baseline		
Quantitative								
Conley et al. Hundert et al.	RCT Follow-up (2 months)	118 55	Evaluate the effectiveness of Honest, Open, Proud-College (HOP-C), a peer-led group-based self-disclosure intervention Evaluate longer-term outcomes of HOP-C at 2-month follow-up	Universities, USA	College students	Self-reported mental health difficulties	20.8 (4.99) 19.20 (1.37)	Female (82.2%) Female (85.45%)
Mulfinger et al.	RCT	98	Evaluate the effectiveness of Honest, Open, Proud (HOP), a peer-led group-based self-disclosure intervention	Clinics, Germany	Adolescents	Self-reported axis I or axis II disorder; moderate level of self- reported disclosure-related distress	15.7 (1.1)	Female (69.3%)
Neiderkronthentaler et al.	RCT	158	Evaluate the effectiveness of an online self-directed video-intervention	Online, Germany	College students	At least two symptoms for DSM-IV criteria for major depressive disorder	Not reported	Female (93.7%), Male (5.1%), Other (1.3%)
Ofoegbu et al.	RCT	60	Evaluate the effectiveness of a group-based video-intervention in schools	Schools for special education needs, Nigeria	Adolescent athletes with special needs	Moderate to severe depression	15.33 (1.73)	Female (40%)
Qualitative								
Lindholm et al.		37	Explore participants’ experiences of the Depression in Swedish Adolescents (DISA) programme	Schools, Sweden	Adolescents	None	–, (12–17)	Female (100%)

Participants from most included studies (n = 3) were recruited based on clinical caseness (i.e., symptom scores above specified thresholds for anxiety or depression). One study by Conley et al. (Conley et al., 2020) took an open-access approach in which college students could self-refer based on felt need. Although cut-offs were not specifically applied for eligibility, the recruited sample comprised 85.5% participants who scored above cut-offs for depression and 69.2% above cut-offs for anxiety. One study recruited a non-clinical sample (Kvist Lindholm, 2015), and one study was a follow up (Hundert et al., 2021).

### Intervention Characteristics

Four self-disclosure interventions were investigated across the six included papers (Table 2). ‘Honest, Open, Proud’ (HOP) was evaluated in three papers (Conley et al., 2020; Hundert et al., 2021; Mulfinger et al., 2018) of which two papers contained results from the same participant cohort assessed at different timepoints (Conley et al., 2020; Hundert et al., 2021).

**Table 2:** Intervention characteristics

Paper	Programme name	Delivery format	Modules	Number of sessions and intervention duration	Intervention access setting	Facilitator
Conley et al. Hundert et al.	Honest, Open, Proud-College (HOP-C)	Peer-led group programme	Three sessions programme including vignettes, role-plays, self-reflection exercises, and group discussions about disclosure, including a consideration of pros and cons of disclosing in different settings, and ways to share one’s story Booster session reviewing intervention materials	Three weekly sessions over 3 weeks, one booster session 2–3 weeks after intervention One booster session; three weeks post intervention	University	Peer facilitators with lived experience
Mulfinger et al.	Honest, Open, Proud (HOP)	Peer-led group programme	German translation of HOP programme; added vignettes about disclosure and social media	Three sessions over 3 weeks	University	Peer facilitators with lived experience
Neiderkronthentaler et al.	[Unnamed]	Video	“Thinking aloud. Jule about her depression”: video featuring lay person with personal experience of depression and suicidal ideation	One session	Online	None
Ofoegbu et al.	[Unnamed]	Video and discussion	Digital stories focused on depression and its management followed by 10–15 discussion	24 sessions over 12 weeks; ~ 30 min each	School	Therapist
Lindholm et al.	Depression in Swedish Adolescents (DISA)	Group-based classroom programme	Cognitive restructuring exercises, self-disclosure in front of classmates	Not reported	School	Teacher

HOP is a peer-led group programme designed to facilitate discussions about the potential impacts of self-disclosure related to mental health problems and to support people in their disclosure decisions. It includes vignettes, role-plays, self-reflection exercises, and group discussions about the risks and benefits of self-disclosure. HOP in the included studies was facilitated by young adult peers with lived experience of mental illness or jointly by a young adult peer and therapist.

The HOP interventions for school and college students (Conley et al., 2020; Mulfinger et al., 2018) considered participants as recipients and disclosers. Intervention contents included exploring stories of other young people’s mental health problems, discussion of the pros and cons of disclosure, and learning to share personal stories including practicing how to tell one’s own mental health story.

The remaining three interventions comprised two video-based interventions and one school-based prevention programme. The first, an online unsupervised video-based intervention featured a filmed narrative about a young person’s lived experience of depression and suicidality (Niederkrotenthaler & Till, 2020). The second was a group video-based intervention based on the principles of Rational-Emotive Behaviour Therapy (REBT) that aimed to decrease dysfunctional beliefs, unhealthy emotions, and problem behaviours by teaching rational alternative beliefs. The intervention was school-based and featured filmed personal stories of depression and its management followed by brief discussion moderated by a special educator (Ofoegbu et al., 2021). The third was Depression in Swedish Adolescents’ (DISA), a group prevention intervention in schools based on cognitive behavioural therapy (CBT), aimed at helping students become more aware about how thoughts and behaviours affect how they feel. The intervention included self-disclosure in front of classmates and was facilitated by teachers (Kvist Lindholm, 2015). The first two interventions (Niederkrotenthaler & Till, 2020; Ofoegbu et al., 2021) focused on participants as recipients while DISA (Kvist Lindholm, 2015) focused on participants as disclosers.

The number of intervention sessions and their duration varied from a single session (Niederkrotenthaler & Till, 2020), to 24 sessions over two months (Ofoegbu et al., 2021). Session duration varied from 30 min (Ofoegbu et al., 2021) to two hours (Mulfinger et al., 2018).

### Data Collection Methods

Relevant quantitative outcomes included self-reported measures of depression and/or anxiety symptoms (Table 3). Mulfinger et. al. collected written qualitative data using a single open-ended question asking about what they liked and disliked about HOP (Mulfinger et al., 2018). More in-depth qualitative data were collected in a study of DISA, which employed focus group discussions and individual interviews with intervention participants. Qualitative data collection in this paper focused on content, form and perceived impacts of the depression programme (Kvist Lindholm, 2015).

**Table 3:** Quantitative results

Paper	Sample size	Control group	Outcome measure	Baseline to post-treatment						Post-treatment to final follow up				
				Intervention M(SD)		Control M(SD)		Effect size	p value	Final follow up duration	Intervention M(SD)	Control M(SD)	Effect size	p value
				Baseline	Post	Baseline	Post				Final follow-up	Final follow-up		
Conley et al.	I = 53, C = 54	Waitlist	CES-D 10	1.66 (0.57)	1.57 (0.67)	1.64 (0.61)	1.53 (0.71)	0.057	–	6 weeks	1.54 (0.67)	1.44 (0.74)	0.003	0.743
			GAD-7	1.66 (0.77)	1.73 (0.78)	1.92 (0.75)	1.79 (0.86)	0.073	–	6 weeks	1.66 (0.75)	1.69 (0.90)	0.017	0.213
Hundert et al.	I = 26, C = 29		CES-D 10	1.74 (0.58)	1.71 (0.63)	1.65 (0.59)	1.56 (0.68)	0.228	–	2 month follow up	1.54 (0.70)	1.39 (0.77)	0.006	0.860
			GAD-7	1.70 (0.73)	1.88 (0.79)	1.96 (0.76)	1.87 (0.82)	0.012	–	2 month follow up	1.57 (0.88)	1.77 (0.85)	0.053	0.252
Mulfinger et al.	I = 49 C = 49	TAU	CES D 10	–	–	–	–	0.12	0.50	–	–	–	0.72	< 0.001
Niederkrotenthaler et al.	I = 81 C = 77	Thematic control video	EDS	–	–	–	–	-	-	6 weeks	3.67 (−)	3.82, (−)	–	< 0.05
Ofoegbu et al.	I = 30 C = 30	Oral stories	BDI-II	35.36 (10.95)	11.43 (3.20)	33.83 (6.31)	38.27 (6.12)	5.50	0.001	6-months post intervention	12.70 (3.09)	36.30 (7.23)	4.24	0.001

*CES-D* Center for Epidemiologic Studies Short Depression Scale 10; *GAD-7* Generalized Anxiety Disorder 7-item Scale; *EDS* Erlangen Depression Scale; *BDI-II*, Beck Depression Inventory- Version II

### Risk of Bias Within Studies

Studies were of variable quality. Lack of blinding of outcome assessors was the most rated source of bias. The qualitative study was rated high quality.

## Synthesis of Results

### What is the evidence for the benefits and potential harms of self-disclosure in interventions aimed at preventing, treating, or managing anxiety and depression among 14–24 years-olds? (Research Question 1)

Among the RCTs that measured impacts for disclosers, an evaluation of HOP (Mulfinger et al., 2018) found that the peer-led group intervention had a large effect on depressive symptoms in a selected sample of school-going depressed adolescents relative to treatment as usual. HOP versus a waitlist control was also evaluated with a self-referring sample of college students and did not show any significant effects on depression (or anxiety) at post-intervention (Conley et al., 2020). However, a small effect on anxiety was found at two-month follow-up (Hundert et al., 2021).

Two video-based intervention studies evaluated self-disclosure from the perspective of recipients. Both studies showed reduced depressive symptoms at post-intervention (Niederkrotenthaler & Till, 2020; Ofoegbu et al., 2021). One study was conducted with participants with symptoms of depression and suicidality and showed an overall beneficial effect for depression but not for suicidal ideation. However, control arm participants who screened positive for moderate-to-severe depression showed a small but significant increase in suicidal ideation. (Niederkrotenthaler & Till, 2020) The second study that evaluated a group video-based intervention delivered to student athletes with special education needs in Nigeria, showed a large effect on depression scores compared with control participants who received an oral storytelling intervention (Ofoegbu et al., 2021).

The qualitative study by Lindholm et al. (Kvist Lindholm, 2015) examined participants’ views about perceived benefits and risks of self-disclosure as part of a classroom-based programme in Swedish secondary schools. Participants identified benefits in terms of stronger interpersonal relations through sharing their private thoughts and feelings with friends or other peers. A similar view was shared by trial participants from Mulfinger et al. (Mulfinger et al., 2018) who especially appreciated the openness, trust and respect of the group sharing experience.

The YPAG highlighted benefits of self-disclosure as including the ‘*helpful release of difficult emotions or experiences’* and *‘the sense of belonging or togetherness’*, especially in group settings (Table 4). Receiving an empathic and non-stigmatising response from recipients of a disclosure narrative, both in person and online, was also described as positively reinforcing.

**Table 4:** YPAG quotes on experiences with and preferences for self-disclosure interventions

Theme	Quotes
Potential benefits	Self-disclosure works for me personally because it helps me put my thoughts into perspective, it feels good to be heard for a change and let all that negativity out It is a helpful release of difficult emotions or experiences and gives me a sense of belonging or togetherness
Potential risks	When I shared my feelings on an online platform, I did feel good. It did have a short-term benefit. But personally, I felt all those feelings again the next day, and the day after as well. So, although I felt good initially, it didn't "fix" anything I think queer individuals would face a lot of difficulty disclosing due to stigma
Settings	I wouldn’t want to disclose in my school or university. It could lead to bullying. Teachers are still not open to these topics and are not accepting of it at all. The environment and the attitudes of the system play an important role in self-disclosure Being able to opt out is important, also being able to be anonymous
Formats	What really stood out to me as helpful was using art or other forms of expression to communicate, and not just words. Asking people to write about their experience is not always ideal because it involves having the skills to write and not everyone can do this I think self-disclosure videos is more intimate and can give people a face, including the non-verbal cues through body language and facial expression even through a screen
Types of content	The languages people’s stories are shared in matters. Also, how many marginalized voices you have included—the more diverse the voices, the more people will be able to relate Struggle does not always lead to recovery, and it is important to showcase difficulties
Mechanisms	When you realise that it’s OK to talk about these things and you are not alone, your stigma is reduced, and it is in turn easier to open up to people or get help Just knowing others have similar experiences

There was no evidence on potential harms from the included studies. To shed light on this area, we consulted YPAG members who indicated that the potential ‘costs’ of self-disclosure included receiving negative or stigmatising responses, especially towards individuals identifying as lesbian, gay, bisexual, transgender, queer, intersex and asexual (LGBTQIA). YPAG members also noted that self-disclosure may not bring about symptom-relief in the short-term, and this might deter some young people. It was felt that interventions should showcase not only recovery stories but day-to-day difficulties and challenges of living with depression or anxiety, emphasising that* ‘struggle does not always lead to recovery, and it is important to showcase difficulties’.*

### For which clinical and demographic subpopulations does self-disclosure appear to be more/less effective? (Research Question 2)

None of the included studies carried out formal moderation analyses of intervention effects. There was indirect evidence that higher distress levels may be associated with stronger effects, based on relative outcomes for two HOP studies. In the first study, Conley et al. (Conley et al., 2020) did not find effects on depression or anxiety outcomes for a self-referring community sample of college students. The second study by Mulfinger et al. (Mulfinger et al., 2018) found significant reductions in depressive symptoms for adolescents recruited from a clinical setting at follow-up assessment but not immediately post intervention.

The YPAG recognised that certain marginalised groups such as young people who identify as LGBTQIA, or those who are restricted by language barriers, and/or face limited access to technology may find it difficult to participate in self-disclosure interventions. Relatedly, they recommended deliberate inclusion of narratives from different genders, languages, and from diverse economic and socially vulnerable youth groups as essential to building relatability.

### In What Contexts do Self-disclosure Interventions Appear to be More/Less Effective? (Research question 3)

There was scarce evidence about the impacts of self-disclosure in different contexts. No studies in this review made comparisons of interventions with different contextual features. In addition, the small number of studies does not allow for drawing of clear inferences about contextual modifiers of effectiveness. It is nonetheless notable that effective interventions were diverse in terms of duration (ranging from a single session(Niederkrotenthaler & Till, 2020) to 24 sessions over two months (Ofoegbu et al., 2021)) and format (video-based (Niederkrotenthaler & Till, 2020; Ofoegbu et al., 2021) and activity and discussion-based (Conley et al., 2020; Hundert et al., 2021; Kvist Lindholm, 2015; Mulfinger et al., 2018)) and in varied settings (schools (Ofoegbu et al., 2021), clinics (Mulfinger et al., 2018) and online (Niederkrotenthaler & Till, 2020)).

Findings from Lindholm et al. (Kvist Lindholm, 2015) provide several qualitative insights into format preferences. Many participants liked spending time in smaller groups that were conducive to sharing private thoughts and feelings and helping to engender mutual acceptance. Disadvantages of the group setting included the risks of bullying and harassment, negative reactions of group members, mandatory participation, and uncertainties about the use of private information. Participants felt that these risks could be mitigated by meeting in small groups, voluntary participation, revision of group composition to include friends or familiar peers, paying attention to how classmates responded to one another, and being clear about how private information is used or shared.

Aligned with these findings, the YPAG agreed that interventions in smaller groups (of six or fewer people) would feel most comfortable and limit negative consequences such as bullying. However, some YPAG members felt that disclosure in educational settings like schools or colleges could have penalising consequences irrespective of group size. Choice and anonymity through ‘*being able to opt out’* and *‘being able to be anonymous’* were identified as important prerequisites to making a disclosure decision. Relational aspects of the disclosure context such as ‘*feeling safe’, ‘non-judgmental’* and *‘an empathetic and understanding audience’* were deemed more important than the physical setting.

Intervention facilitators across the studies varied and included peer facilitators with lived experience (Conley et al., 2020; Mulfinger et al., 2018), therapists (Ofoegbu et al., 2021) and teachers (Kvist Lindholm, 2015). Participants from one study reported viewing peer facilitators with lived experience as inspiring ‘role models’ and contributing to building trust in the group. They also reported finding the workbook provided as part of the intervention materials valuable, highlighting realistic scenarios of self-disclosure presented as especially useful (Mulfinger et al., 2018).

The YPAG agreed that in addition to mental health professionals, peer facilitators or ‘programme ambassadors with lived experience’ of anxiety or depression could serve as intervention facilitators. They also expressed a clear preference for the use of video-based formats which offer *‘non-verbal cues through body language and facial expression even through a screen’* and non-verbal formats such as art.

### What are the putative mechanisms by which self-disclosure interventions influence outcomes in the target population? (Research Question 4)

The included quantitative studies did not incorporate mediation analyses and the qualitative study did not explicitly examine mechanisms of self-disclosure.

The YPAG suggested several pathways through which self-disclosure may impact outcomes. They reported that self-disclosure can lead to reduced mental health symptoms by ameliorating social isolation (*‘knowing others have similar experiences’).* Self-disclosure was also considered to be an important outlet for both making sense of and expressing difficult thoughts or feelings, as well as helping to identify potential options to solve stressful problems, which may help with resolving stressors that in turn affect depression and anxiety.

The role of stigma reduction as part of the disclosure process in facilitating help-seeking was also recognised; ‘*when you realise that it*’*s OK to talk about these things and you are not alone, your stigma is reduced, and it is in turn easier to open up to people or get help*’.

## Discussion

This review synthesised evidence on self-disclosure interventions for anxiety and depression among adolescents and young adults, with the intention to examine benefits and potential harms, putative moderators, and mechanisms through which self-disclosure can affect anxiety and depression outcomes.

Overall, we found that self-disclosure can improve depressive symptoms for young people who personally share or learn about experiences of depression; we did not find evidence of impacts on anxiety. There was no peer-reviewed evidence available on potential harms and no results available from formal moderation or mediation analyses. Gaps in the quantitative evidence base were supplemented by qualitative findings and insights from a lived experience panel. These sources highlighted preferences for group formats due to the togetherness they foster; potential negative effects like bullying; and recommendations for the inclusion of diverse depression and anxiety narratives showcasing different genders, languages, and vulnerable adolescents and young adults’ experiences into interventions. The potential role of adolescents and young adults with lived experience of anxiety and depression in delivery of interventions was also highlighted.

Although this review includes a very small number of peer-reviewed studies, it suggests that self-disclosure may be of value in the context of interventions intended explicitly to reduce depression for those already showing symptoms.

Among the included interventions implemented, it appears that those that aimed to build specific disclosure-related skills such as ‘Honest, Open, Proud’ (HOP) (Conley et al., 2020; Mulfinger et al., 2018) may not reduce psychological distress immediately. However, this may be because these interventions are not designed to improve mental health outcomes directly or do not allow participants to put newly learned skills into practice.

A notable finding in our review was that of the three studies which showed positive effects for depressive symptoms, two studies were conducted in supervised face-to-face settings with counsellor or peer facilitator support (Mulfinger et al., 2018; Ofoegbu et al., 2021) while the third study was conducted online with no counsellor support (Niederkrotenthaler & Till, 2020). These findings contrast with insights from a recent meta-analysis of digital interventions for adolescents with anxiety and depression which suggested that interventions without regular and/or high levels of supervision or therapist involvement may not be effective in causing clinically detectable levels of change (Garrido, et al., 2019). These results suggest that self-disclosure interventions involving personal experiences may compensate for preferences for human interaction in psychological interventions (Bucci et al., 2016; Garrido, et al., 2019).

Moreover, the YPAG shared preferences for the privacy and anonymity that online or social media platforms offer. At the same time, they cautioned that online experiences could be mixed, with potential for both positive and negative effects. This insight is consistent with recent findings on the mixed experiences of social media by young people (Naslund et al., 2020; Seabrook et al., 2016; Taniguchi & Glowacki, 2021).

Qualitative findings and YPAG insights showed that group-based interventions combining activities such as role-plays, self-reflection exercises, and group discussions about disclosure conducted in a supportive group setting were perceived as helpful. This insight is aligned with research from both high and low-and-middle income settings that shows group interventions, especially those which are short-term and address emotional or behavioural difficulties in an inclusive and supportive manner can have positive impacts on social and emotional wellbeing while also offering practical advantages in terms of cost, time, and manpower efficiency, and offering young people the opportunity to work alongside peers with similar difficulties (Cheney et al., 2014; Das et al., 2016; Ninan et al., 2019). Additionally, research on group-based belonging and social identity processes shows that building social identification within a group can play an important role in a range of health outcomes that extend outside of the group setting, even offering protective value against depression symptoms (Cruwys et al., 2021; Steffens et al., 2021).

The bi-directional relationship of stigma with self-disclosure (i.e., the impact of self-disclosure on reducing stigma and of stigma reduction on facilitating self-disclosure) was indicated through studies which had a joint focus on stigma reduction and building self-disclosure skills (Conley et al., 2020; Hundert et al., 2021; Mulfinger et al., 2018). Although existing reviews have not examined the role of self-disclosure directly on outcomes related to adolescent or young adults’ depression and anxiety, Corrigan et al. showed that contact-based interventions with persons with mental illness may be an especially helpful way to reduce public stigma for both adults and adolescents (Corrigan, 2012). Further, aligned with findings from this review that suggest both stigma reduction and building skills to disclose may be crucial first steps to supporting young people in making safe disclosures, Scior et al. suggest that safe and meaningful self-disclosure ultimately support recovery processes through generating hope, reducing shame and enhancing self-esteem (Scior et al., 2020). Further, reducing self-stigma about receiving a mental health diagnosis and being able to make empowered decisions about disclosing a diagnosis have been shown to be effective strategies in supporting adolescents' recovery from serious mental illness (Dubreucq et al., 2021).

### Limitations and Strengths

This review has several limitations including the small number of studies and small number of anxiety studies relative to depression studies; low representation of peer-reviewed studies from low-or middle-income countries; majority female participants across studies; and relatively few young adults over 18 years of age. No quantitative studies included a process evaluation. Included interventions were variable in terms of format, participants' context, and nature of delivery, making it difficult to make comparisons and draw conclusions. Although included studies reported on outcomes such as stigma stress, disclosure-related distress, help-seeking, and attitudes to disclosure which qualitative insights showed may be important intermediate outcomes, this review did not examine any other outcomes aside from depression and anxiety. Finally, due to the risk of bias in most studies included in this review, results should be interpreted with caution.

Nevertheless, this report provides the first systematic review of self-disclosure interventions for anxiety and depression among adolescents and young adults. The involvement of and incorporation of lived experience insights from adolescents and young adults, especially from a low-and-middle-income country, is a key strength of this study and helped to corroborate findings from the included studies and offer additional perspectives to help fill gaps in the available evidence. The lived experience contributions to this review are aligned with previous participatory reviews involving young people which include consultative workshops to draw on the perspectives of young people when interpreting and reflecting upon findings (Oliver et al., 2015).

### Implications

Of importance in our findings is the delivery of interventions by non-specialists such as peers with lived experience (Conley et al., 2020; Mulfinger et al., 2018) and teachers (Kvist Lindholm, 2015). There is already a growing understanding of the benefits of involvement of persons with lived experiences in mental health care by increasing awareness, reducing stigmatization, and improving access to treatment and services (CCSA) & C.C.o.S.A., 2013; Vojtila et al., 2021;). Findings from this review also support exploring the involvement of adolescents and young adults with lived experiences of depression or anxiety in building capacity within community health systems.

Aligned with observations by Wolpert et al. on the disconnect between clinical research and understanding of the mechanisms of change underpinning ‘active ingredients’ for depression and anxiety among adolescents and young adults (Wolpert et al., 2021), more research on effects and mechanisms of self-disclosure is needed.

The YPAG prioritised their top future research priorities for self-disclosure interventions (Table 5) which included research on underpinning mechanisms of self-disclosure, whether self-disclosure can help prevent anxiety of depression, the role of self-disclosure for members of vulnerable groups, the role of lived experience peer-facilitators and barriers and facilitators to disclosure in different interventions settings.

**Table 5:** YPAG ranking of future research questions examining self-disclosure impacts on depression and/or anxiety

Ranking	Research question
1	What are the underpinning mechanisms for why self-disclosure interventions work?
2	Can self-disclosure interventions prevent depression and/or anxiety in adolescents/young adults aged 14–24?
3	How do specific vulnerabilities or marginalization related to gender identity interact with how adolescents/young adults with depression or anxiety self-disclose?
4	How can people surrounding adolescents/young adults with depression or anxiety help to encourage safe self-disclosure?
5	Does listening to self-disclosure from peers lead to an increase in help-seeking among adolescents/young adults?
6	Do outcomes of self-disclosure programmes facilitated by professional therapists or adolescents/young adults with lived experience differ? If so, how?
7	What are specific barriers and facilitators for adolescents/young adults disclosing their depression and/or anxiety in different settings? (Education, workplace, online, etc.)
8	What types of self-disclosure formats are most effective at reducing depression/anxiety for recipients participating in a prevention programme?
9	In which setting is self-disclosure more helpful- group or 1:1 or online?
10	Who benefits most from self-disclosure interventions? Adolescents/young adults with anxiety or depression or mixed anxiety and depression?

## Conclusions

This review builds on a limited existing understanding of the impacts of self-disclosure for depression or anxiety outcomes for young people aged 14–24 years. Review findings and lived experience panel synthesis show that self-disclosure interventions can be effective for adolescents and young adults with depression when delivered online or in person in groups in supervised settings. This review also highlights the role that lived experiences of depression and anxiety can play in intervention contents as well as delivery via lived experience facilitators.

More research is needed with different groups of young people across different age groups, genders, anxiety and/or depression caseness; and in different contexts, such as educational settings or online and in more diverse contexts including low-and-middle-income settings; and with different delivery agents including non-specialists such as adolescents or young adults with lived experience. Finally, research is needed to understand the specific mechanisms underpinning self-disclosure for adolescents or young adults with anxiety and depression to better understand how self-disclosure interventions are most beneficial (and how to mitigate potential harms).

## Appendix A: Database search keywords

**Table:**

Element	Search terms and Boolean operators
Sample age	(Adolescen* OR teen OR young OR youth OR student OR child*) AND
Phenomenon of interest	(Self-discl* OR disclos* OR “distress-disclosure” OR narrative* OR stor* OR personal experience* OR "lived experience" OR account*) AND
Sample condition	(Depress* OR anxi*) AND
Research setting	(Therap* OR psychotherap* OR intervention* OR treat* OR prevent* OR app OR application OR program* OR in-patient OR out-patient OR clinic OR online OR social media OR social network OR web OR internet) AND
Research design	(Trial OR pilot OR feasibility OR pre-post OR qualitative OR mixed method* OR interview OR focus group OR experimental OR evaluation)

## Appendix B: Study inclusion criteria

**Table:**

Element	Inclusion criteria
Participants	Inclusion: •Interventions focused on young people aged 14–24 years either as disclosers or as recipients of self-disclosure. (Interventions themselves may include self-disclosure by other age groups provided that the recipients are within the 14–24-year-old age bracket.) •Disclosers are required to have a current or past experience of depression and/or anxiety based on (a) a clinical diagnosis or formulation made by a mental health professional, (b) elevated symptoms confirmed by a standardized assessment tool, or (c) subjective self-report •In the case of preventive interventions, the recipients of personally disclosed information about anxiety and depression may be young people who have not (yet) experienced either condition Exclusion: •Studies where a majority of participants fall outside the 14–24-year-old age cohort
Interventions	Inclusion: •Interventions employing intentional self-disclosure, defined as revealing personal information about lived experiences of anxiety or depression, with the goal to prevent onset of, treat, manage, or prevent relapse of anxiety and/or depression for the disclosers and/or recipients of such information Exclusion: Studies focused on therapists’ self-disclosure were not included
Study design and context	•For comparative trials, the outcomes of self-disclosure interventions are compared against any other comparator or control (which could include alternative delivery formats for self-disclosure) •Outcomes for uncontrolled pre-post evaluations of self-disclosure interventions •Studies from any geographical location, encompassing any health, community, educational or online setting
Outcomes	For the analysis of potential benefits: •The primary quantitative outcomes of interest are improvements in depression and anxiety, measured by validated symptom-based or diagnostic instruments •We are also interested in qualitative reports of symptomatic/diagnostic and functional improvements (e.g., in interpersonal, occupational, and educational domains) For the analysis of potential harms: •Quantitative outcomes of interest are deteriorations in depression and anxiety, measured by validated symptom-based or diagnostic instruments, and serious adverse events defined according to the original study protocols (where reported) •We are also interested in qualitative reports of negative effects on self (e.g., symptoms, functioning, dependency) or others (e.g., stigma experienced from family, friends, peers, community members) For the analysis of mechanisms: •Quantitative outcomes of interest are assessed mediators of intervention effects on anxiety and depression •We are also interested in qualitative reports of how intervention content and materials are taken up and used by recipients/disclosers in self-disclosure interventions; and accounts of how recipients/disclosers describe the ensuing intervention mechanisms and pathways to beneficial outcomes (and potential harms)

## Appendix C: Young People’s Advisory Group (YPAG) summary of self-disclosure projects

### Project Background

Our research project, which was commissioned by the Wellcome Trust, examined the benefits and potential harms of self-disclosure in interventions aimed at preventing the onset of, or treating, managing or preventing relapse of anxiety and depression among 14–24 years-olds. This project systematically reviewed the evidence for self-disclosure as an active ingredient in the management and treatment of adolescents and young adults with anxiety and depression in collaboration with a panel of seven young people with lived experience who comprised the ‘Young People’s Advisory Group’ (YPAG). Over eight weeks, the YPAG worked independently with support from the research team on conducting a rapid online scoping and listing of global youth-led and youth-focused initiatives that use or promote self-disclosure as a strategy for the prevention, management or treatment of anxiety and depression among adolescents and young adults.

### Composition of the Young People’s Advisory Group (YPAG)

Our Young People’s Advisory Group (YPAG) comprised seven individuals aged 19–25 years with lived experiences of depression and/or anxiety. Five of these individuals identified as female, and two identified as male, and they belonged to varied cultural backgrounds and geographic states across India.

They were recruited via Sangath’s national youth mental health programme in India, *It’s Ok To Talk,* to work with the research team over six web-based sessions. These sessions consisted of an orientation, training in participatory review methods and systematic review processes, presentations of research conducted by the YPAG, discussions about challenges they were facing in their research, and discussions on their lived experience of self-disclosure.

### Collaborative Research Activity Methods

The YPAG had two primary tasks: (1) to review the research protocol and comment on its findings, and (2) to conduct a scoping of global self-disclosure programmes either led by or focused on adolescents and/or young adults with depression or anxiety. Specific search criteria included projects related to self-disclosure, projects led by or focused on young people (aged 14–24 years), and projects focussed on the treatment, prevention or management of depression or anxiety.

The YPAG was advised to search for sources such as programme-or-organisational websites, programme reports, blogs or social media pages, and global advocacy networks. Scoping was conducted by the YPAG on Google, Google Scholar, mental health aggregators like MHIN (Mental Health Innovation Network), and social media platforms like Instagram. These searches were undertaken using keywords taken from the research protocol document.

The research team provided regular support to the YPAG teams via email, Zoom meetings and WhatsApp check-in calls. They reviewed the YPAG’s final findings to ascertain if inclusion criteria had been met and undertook a gap-filling exercise to locate any programmes that the YPAG might have missed.

### Summary of Findings

#### Regional Breakdown

Thirty six self-disclosure programmes that met the search criteria were identified primarily in Australia, Canada, China, India, New Zealand, Pakistan, the UK, and the USA. Very few programmes were found in Africa and no programmes that matched our criteria were found in South and Central America or Europe.

#### Target Population

Seventy one of the 36programmes found did not have any specific focus on young people, but catered to all age-groups, and only three programmes specified the age group of the target population (as 18 + years, 16–24-year-olds, etc.). Many programs also used terms like “young adults” and “young people” to showcase their target population. All the programmes included self-disclosure stories of young people in the age group 16–30 years, and all programmes identified catered to all genders.

#### Self-disclosure Formats

Five different kinds of self-disclosure formats were found, where *format* refers to the various modes of expression of self-disclosure content. The most common self-disclosure format found was blogs, with collections of text-based stories. Many programmes used multimedia storytelling formats, inviting self-disclosure content in the form of videos, video-based interviews, photographs, paintings, watercolour images and poetry. Social media projects like Instagram’s #HereForYou Campaign were found to encourage users to open up about their own struggles with mental health through various media formats and join a global conversation by using the hashtag #HereForYou. Two programmes by the National Alliance on Mental Illness (NAMI), *You are not alone* and *OKtotalk*, invited people to talk and self-disclose through a Tumblr board, and provided readers access to support, advice and lived experience stories of individuals with mental illness via these online forums.

#### Depression and/or Anxiety Focus

Thirty two programmes identified focussed on both anxiety and depression, and the remaining four focussed on either anxiety or depression. Many programmes disseminated self-disclosure content focussed on varied mental health concerns, such as *This Is My Brave,* which has produced over 75 shows in cities across the United States featuring almost 875 storytellers sharing personal stories on overcoming depression, anxiety, bipolar disorder, PTSD, OCD, postpartum depression, alcoholism, substance use disorder and more. UK’s *No More Panic* provided access to forums, Chat Rooms, and member’s stories for sufferers and carers of people with panic, anxiety, phobias, and obsessive–compulsive disorder (OCD). *To Write Love on Her Arms*, from the United States, has published a blog dedicated to presenting stories of hope and providing help to people struggling with depression, addiction, and suicide.

#### Programme Goals

Some programmes encouraged users to share their lived experience stories of anxiety and depression. The mission of *This is my Brave* was to empower individuals to put their names and faces to their stories of recovery from mental illness and addiction. A few self-disclosure programmes helped users build connections, make friends, and meet like-minded people who would understand and empathise with their struggles, such as Depression UK’s *Friendship & Pen Friend Scheme*, wherein members could write to fellow ‘Helper-Sufferers’ to share their troubles, success stories, strategies of coping etc. A few programmes used self-disclosure to end stigma or discrimination, or increase awareness about mental health such as The *Make it Okay* and the* #VoicesOfHope* campaigns.

#### Achievements

The programmes helped their beneficiaries disclose their mental health concerns, encouraged new friendships, and helped them find communities of like-minded people who could empathise with them. The programmes also enabled conversations around mental health by providing people a safe space for self-disclosure. This, in turn, would lead to stigma reduction and encourage treatment-seeking.

#### Gaps Identified

Some of the programmes identified did not have adequate social media marketing, and that made it difficult for people to discover these projects online. A few programmes, like NAMI’s, You are not alone and OKtoTalk, were only accessible to people who use social media platform Tumblr, and similarly with Instagram’s #HereForYou campaign– if one did not have an account on these social media platforms, one could not access these communities of support. Moreover, while online self-disclosure could be beneficial, some adolescents and young adults might need a *face-to-face connection* to feel comfortable enough to share their lived experiences.

#### Challenges Faced

It was challenging and time-consuming to identify programmes that fit the inclusion criteria provided. Many global programmes did not have sufficient social media presence, and thus, searching on Instagram/other social media platforms was not very helpful. Country specific programs were hard to find due to social media algorithms providing only location-based results. Therefore, VPNs (Virtual Private Networks) were used to solve this issue. Language barriers existed while searching for programmes in South America, since a lot of the initiatives on Instagram were found using Spanish as their main language. In countries with high stigma associated with mental illness, such as South Korea, self-disclosure programs were found to be low in numbers. Most of the programmes identified by the YPAG have not been evaluated for their impacts, and the achievements listed above have been compiled based on information available on the project’s website or social media channels.

### YPAG Insights from Web-sessions

#### Context of Self-disclosure

Self-disclosure programmes that invited in-depth stories about the participants’ life events, and not only about their experiences of anxiety or depression were considered helpful. Videos were encouraged because people’s faces had the potential to offer non-verbal cues which text cannot. Similarly, artistic, and poetic depictions were considered easier as formats of disclosure. They also recommended interventions which did not focus on positivity and recovery, but also elaborated on the struggles caused by depression and anxiety.

#### Barriers to Self-disclosure

It was felt that young men and boys struggle to speak about their struggles and would benefit from self-disclosure to a supportive audience. Similarly, queer individuals would face difficulty while sharing their experiences due to stigma. Unsympathetic audiences who either did not understand or harassed the disclosers were considered the greatest barriers to self-disclosure.

#### Enablers of Self-disclosure

The panel felt that active listening and a supportive audience could greatly encourage self-disclosure. Small-scale peer-led discussion forums led by someone who has lived experience could help participants open up, and watching a celebrity talk about their own depression experience could help remove stigma.

#### Why Does Self-disclosure Work?

The YPAG shared that self-disclosure helped them feel unburdened, as their thoughts and energy that are concentrated inside find an external channel or “release” when they are talking about their experiences. Self-disclosure could help both recipients as well as disclosers feel that they are not alone, and it is comforting to feel that other people have had similar experiences. This provides a sense of belonging and togetherness. Self-disclosure also helps disclosers put their thoughts into perspective and helps them make sense of their thoughts and feelings.
